# Functions of Tooth Pulp

**Published:** 1890-02

**Authors:** Alice Sherman


### Functions of Tooth Pulp.

BY ALICE SHERMAN.

Between the sixth and tenth week of embryonic life twenty minute elevations arise in the primary dental grove just behind the soft tissues which subsequently form the lips. These develop into the pulps of the temporary teeth, but the pulp of every tooth has its origin in a papilla, and the study of its functions may well commence with the papillary stage in the development of teeth.

The delicate papilla continues growing ; it is isolated by partitions and by a depression or follicle. By the end of the sixteenth week the rising of partitions and the growing toward each other of the enamel organ and the papilla completely enclose the latter and produce the saccular stage.

What was originally fine fibrous tissue with numerous embryonic cells becomes differentiated into odontoblasts. These send toward the periphery minute processes known as Tome's fibrils, each of which is surrounded by a strong sheath. By a process of reciprocal adaptation the overlying enamel organ and the papilla give *form* both to dentine and enamel.

The pulp is now a soft structure of a shape and size corresponding to the dentine of the complete tooth. Blood vessels, nerves, and possibly lymphatics can be demonstrated in its structure. Just upon the tips of the cusps, at the termination of the dentinal fibrils, the formed material becomes calcified and the first dentine is deposited. Before eruption, and after, the calcification progresses until the peripheral dentine of the crown and entire root is elaborated.

There being no need for further *extensions*, like a wise builder, the "pulp," for that is the name our little "papilla" has assumed—just as a boy changes his childish name to something more blunt and forcible when his growth is completed—turns its attention to the preservation of the structure and the finishing of the interior.

Its nutriment and its warmth come through three principal pipes, called arteries, and by most circuitous ways, and smaller tubes are distributed throughout the tooth structure. The waste is removed by as many mains or veins. Sometimes appeals to its charity is made, and the pulp requested to nourish its neighbor "cementum." Then by a system of peculiar cut-offs, called canals and canaliculi, the pulp turns part of its pabulum into its neighbor's house.

An architect looks after the sanitary regulation of a building lest the inmates die, the structure becomes foul, and the Board of Health condemn it; so the pulp finds employment in maintaining the purity of its home, the vigor of its own life and saving the tooth from exfoliation.

No modern home is complete without communication by telegraph and telephone with local points and the outside world. The pulp receives and gives impressions by means of nerves and a transmitting system we do not understand.

This is not all the pulp has to do. It must go on finishing the interior of its house, adding a little more here, a little there, to strengthen its walls. If there were no destructive agents it would have a less arduous life, but sometimes a message is received warning it to put an indestructible wall in some position for a fire—decay—is near at hand. Its activity increases and a zone of resistance is made.

Occasionally some other force almost wears through its walls and a solid wing is made as a protection. This is of a hard, translucent substance known as secondary dentine.

Do you think the pulp has no work to do? From the beginning of its life, almost from the beginning of the life of the individual, until its disease or death it is one of the most active little organs in the human anatomy.

